# Comparative effects of six exercise modalities on global cognitive function in Alzheimer’s disease, Parkinson’s disease, and mild cognitive impairment: a systematic review and network meta-analysis

**DOI:** 10.3389/fneur.2026.1877537

**Published:** 2026-09-25

**Authors:** Xiaolin Li, Haonan Qi, Xiaoqi Zhang, Xiaoqiang Jin, Jiawei Xu, Jian Ma

**Affiliations:** 1 Department of Physical Education and Training, School of Physical Education, Hebei Normal University, Shijiazhuang, Hebei, China; 2 Department of Chinese Traditional Physical Education, School of Physical Education, Hebei Normal University, Shijiazhuang, Hebei, China

**Keywords:** Alzheimer’s disease, exercise, global cognitive function, mild cognitive impairment, network meta-analysis, Parkinson’s disease

## Abstract

**Background:**

Alzheimer’s disease (AD), Parkinson’s disease (PD), and mild cognitive impairment (MCI) are commonly accompanied by cognitive decline. Although exercise is recommended as a non-pharmacological strategy, the comparative effects of different exercise modalities on global cognition remain uncertain.

**Objective:**

To compare six exercise modalities—aerobic exercise (AE), mind–body exercise (MBE), resistance training (RT), multi-component exercise (MCE), flexibility training (FT), and functional training (FTr)—for global cognitive function in people with AD, PD, or MCI, and to explore dose-related associations.

**Methods:**

PubMed, Embase, the Cochrane Library, and Web of Science were searched from inception to January 26, 2026. Randomized controlled trials (RCTs) were synthesized using a frequentist random-effects network meta-analysis (NMA) with standardized mean differences (SMDs) and 95% confidence intervals (CIs). Treatment rankings were summarized using SUCRA. Subgroup analyses, meta-regression, diagnosis-related sensitivity analyses, and CINeMA certainty assessments were also performed.

**Results:**

Twenty-eight trials involving 1,993 participants were included. Compared with control group (CG), AE [SMD = 0.47, 95% CI (0.18, 0.77)], MBE [SMD = 0.79, 95% CI (0.46, 1.11)], RT [SMD = 1.50, 95% CI (0.77, 2.23)], MCE [SMD = 0.83, 95% CI (0.26, 1.40)], and FTr [SMD = 0.83, 95% CI (0.13, 1.53)] improved global cognition, whereas FT showed no clear effect [SMD = -0.20, 95% CI (−1.27,0.87)]. RT ranked first, followed by MCE, FTr, MBE, AE, and FT. Heterogeneity was substantial (τ^2^ = 0.23; I^2^ = 80%), although no statistically significant global incoherence was detected. RT remained first after the exclusion of AD, PD, or MCI studies. Studies lasting more than 12 weeks, delivered 3–5 times per week, and using sessions of 31–60 min showed favorable pooled effects, but subgroup interaction tests and meta-regression were not significant. Certainty of evidence ranged from low to very low.

**Conclusion:**

AE, MBE, RT, MCE, and FTr may improve global cognition in people with AD, PD, or MCI. RT showed the largest effect estimate and the most favorable ranking. However, substantial heterogeneity and sparse evidence for several modalities limit firm conclusions. Exercise selection should be individualized, and dose-related findings require confirmation in adequately powered comparative trials.

## 1 Introduction

The growing global burden of neurodegenerative disorders and related prodromal cognitive states is reshaping public health priorities (1). Alzheimer’s disease (AD), Parkinson’s disease (PD), and mild cognitive impairment (MCI) affect the health of tens of millions globally (2), and their overall burden is anticipated to increase with an aging population over the next thirty years (3). More importantly, these conditions do not always occur in isolation and often coincide in older individuals (4, 5). Pathological changes associated with AD are commonly observed in PD patients (6), while MCI often serves as a transitional stage that can precede or accompany the development of neurodegenerative disorders (7, 8). MCI was included as a clinically relevant prodromal cognitive state because it frequently precedes dementia-related neurodegenerative disorders and shares cognitive outcomes commonly evaluated in exercise research. Existing neurorehabilitation frameworks are still primarily structured around models that focus on specific diseases, with AD research concentrating on cognitive reserve (9, 10); rehabilitation in PD focuses on managing motor symptoms (11, 12), while interventions for MCI often aim at risk assessment and predicting progression (13, 14). This diagnosis-specific organization has limited direct comparison of potentially shared biological pathways and exercise effects across AD, PD, and MCI. Evidence on similar exercise interventions has therefore accumulated largely in parallel, often through small trials with short intervention durations and heterogeneous findings, and has rarely been integrated within a single comparative framework (15–17).

Exercise, as a low-cost and accessible non-pharmacological intervention, has been widely shown to improve cognitive function (18, 19). However, exercise is not a homogeneous entity: aerobic exercise (AE) involves sustained cardiovascular activation, promoting cerebral blood flow and neurogenesis (20, 21); mind–body exercises (MBE) train executive function and attention networks by combining motor and cognitive dual-tasking (22, 23); resistance training (RT), defined by concentric muscle contractions, activates the muscle-brain signal axis (24, 25); multi-component exercise (MCE), FT, and FTr target various physiological adaptations (26, 27). Previous evidence syntheses have generally focused on individual diagnostic populations. NMA in MCI have compared exercise modalities and explored intervention dose (28–30), whereas reviews in PD have primarily evaluated exercise effects within the context of motor and non-motor rehabilitation (12, 31). Reviews in AD or dementia have also compared exercise modalities, intervention doses, or exercise with usual care and other control conditions (32, 33). Huang et al. (28) additionally synthesized exercise interventions across MCI and dementia. However, these reviews differed in population scope, outcome selection, intervention classification, and analytical objectives. To our knowledge, no previous review has used a single predefined network to compare the same six exercise categories for global cognitive function across AD, PD, and MCI while explicitly examining the implications and limitations of cross-condition evidence synthesis. Public health and rehabilitation guidance generally supports physical activity and structured exercise for older adults and people living with chronic conditions, disabilities, or neurological conditions (34, 35). However, the comparative cognitive effects of specific exercise modalities remain uncertain. Conventional pairwise meta-analyses are limited to intervention comparisons represented directly in the available trials, whereas NMA can integrate direct and indirect evidence across multiple interventions (36, 37). The validity of an NMA, however, depends on the comparability of important effect modifiers across treatment comparisons (36, 37). This issue is particularly relevant when evidence from AD, PD, and MCI is combined because AD and PD have distinct disease mechanisms, whereas MCI is a clinically and etiologically heterogeneous syndrome (38–40).

Accordingly, we conducted a systematic review and NMA to compare the effects of six exercise modalities on global cognitive function in people with AD, PD, or MCI, summarize their relative ranking probabilities, and explore associations between intervention dose and cognitive outcomes. Given the inclusion of clinically distinct diagnostic populations, we also assessed the distribution of potential effect modifiers and examined the robustness of the network estimates through diagnosis-related sensitivity analyses. We hypothesized that structured exercise interventions would improve global cognitive function compared with control conditions and that their relative effects would vary across the six exercise modalities. In exploratory analyses, we further hypothesized that intervention duration, frequency, and exercise time would be associated with the magnitude of cognitive effects.

## 2 Methods

### 2.1 Protocol and registration

Following the PRISMA-NMA2020 guidelines and the Cochrane Handbook, this study conducted a systematic review of interventions (41). The protocol was registered with PROSPERO (CRD number: CRD420261291814). The PRISMA-NMA checklist is provided in Supplementary material 1.

### 2.2 Data sources and search approaches

A comprehensive search was conducted in the PubMed, Embase, the Cochrane Library, and Web of Science databases from their inception until January 26, 2026. Searches mainly centered on the following medical subject headings (MeSH) and free words: Exercise, Cognitive function, Randomized controlled trials (RCTs), PD, AD, MCI. Detailed search methods are offered in Supplementary material 2.

### 2.3 Eligibility criteria

Inclusion criteria were as follows:

*Population:* Adults diagnosed with AD, PD, or MCI according to established clinical, research, or validated study-specific diagnostic criteria. Studies involving mixed populations were eligible only when data for an eligible diagnostic group could be extracted separately or when the entire sample met the prespecified eligibility criteria.

*Intervention*: Exercise alone as the sole intervention.

*Comparisons*: Eligible comparators included no-exercise control conditions, such as usual care or wait-list control, and another eligible exercise modality.

*Outcomes*: At least one validated scale was used to assess cognitive function. Examples include the Mini-Mental State Examination (MMSE) (42), the Montreal Cognitive Assessment (MoCA) (43), etc.

*Study design*: RCTs.

Exclusion criteria were as follows:

(1) Incomplete data precluded subsequent data extraction;
(2) Studies combining exercise with a non-exercise therapeutic co-intervention that was not applied equally across comparison groups were excluded;
(3) Non-English literature;
(4) Systematic reviews, meta-analyses, abstracts, protocols, conference papers, letters, and duplicated articles.

### 2.4 Exercise types

Based on the American College of Sports Medicine (ACSM) Guidelines (44), the Physical Activity Guidelines for Americans (45), and previous systematic reviews (28, 46, 47), exercise was defined as: planned, structured, and repetitive bodily movement done to enhance or sustain one or more elements of physical fitness. Six categories of exercise interventions were defined. The interventions and their definitions are outlined in Table 1. Before study selection was completed, operational classification rules were developed for each intervention node. Interventions were categorized according to their primary training objective, dominant exercise component, and reported allocation of training time. Programs containing two or more substantive exercise components were classified as MCE. Functional exercises were classified as FTr only when the principal objective was the practice of movement patterns directly related to activities of daily living rather than progressive muscle overload alone.

**Table 1:** Treatment nodes included in the NMA.

Type of exercise	Definition	Examples
Aerobic exercise (AE)	AE refers to physical activities that increase heart rate and breathing while engaging large muscle groups in rhythmic motions.	Running, swimming, cycling, and walking, etc.
Mind–body exercise (MBE)	MBE centers on movements that enhance both mental and physical well-being. These exercises combine physical activity with mindfulness, balance, and relaxation techniques.	Yoga, Tai Chi, Pilates, and Qigong, etc.
Resistance training (RT)	RT involves exercises where muscles are exerted against a force, helping to increase strength, endurance, and muscle mass. It can involve the use of free weights, machines, or bodyweight.	Weightlifting, bodyweight exercises like push-ups, and utilizing resistance bands, etc.
Multi-component exercise (MCE)	MCE programs combine various categories of exercises to enhance many aspects of fitness, including strength, endurance, flexibility, and balance.	A fitness class that includes aerobic activity, strength training, and balance exercises, etc.
Flexibility training (FT)	FT involves exercises that enhance the range of motion of muscles and joints, helping to enhance flexibility and decrease injury risks.	Static stretching, dynamic stretching, and foam rolling, etc.
Functional training (FTr)	FTr involves exercises that enhance the ability to perform everyday activities. It centers on movements that mimic general tasks, increasing balance, coordination, and strength.	Squats, lunges, and exercises utilizing kettlebells or stability balls that engage core muscles, etc.

### 2.5 Data selection and extraction

The retrieved articles were loaded into EndNote 20 software to remove duplicate records. Afterward, two reviewers independently assessed the articles. Disagreements were resolved through discussion and, when necessary, consultation with a third reviewer.

The following information was obtained from the included articles: basic details (authors, publication year, country, participant age, sample size and type of disease), trial features (type of exercise, duration of exercise, frequency of exercise and exercise time), and outcome measures (assessment methods, effect-size data, such as mean and standard deviation).

### 2.6 Risk of bias assessment

In this study, two researchers analyzed risk of bias using the Cochrane Risk of Bias 2 tool (RoB-2) across five domains: randomization process, deviations from the intended interventions, missing outcome data, outcome measurement, and selection of reported outcomes (48). Based on the findings, articles were grouped as having low risk of bias (all domains analyzed as low risk), some concerns (at least one domain raising concerns but none categorized as high risk), or high risk of bias (at least one domain recognized as high risk). Disagreements were resolved through consultation with a third researcher.

### 2.7 Statistical analysis

Analyses were performed using Stata 18.0 with the mvmeta, network, metareg, and metan packages. For all outcomes, we carried out a NMA within the frequentist framework.

Changes in cognitive outcomes were treated as continuous variables measured with different scales. Hence, standardized mean differences (SMDs) with 95% confidence intervals (CIs) were reported. All cognitive outcomes were oriented so that higher values represented better cognitive performance. Scores from tools in which lower values indicated better performance were multiplied by −1 before effect-size calculation. For comparisons between an exercise intervention and a control condition, a positive SMD therefore indicated a greater improvement in global cognitive function in the exercise group. When a study reported more than one eligible global cognitive measure, the outcome specified as primary by the study authors was selected; when no primary outcome was specified, a prespecified hierarchy was applied, prioritizing MoCA, MMSE, and then other validated global cognitive instruments. The post-intervention assessment closest to the end of the supervised intervention period was used. Change scores were preferred when available. When change-score standard deviations were unavailable, they were calculated using reported data with alternative coefficients examined in sensitivity analyses. Because clinical and methodological heterogeneity was expected across diagnostic populations, exercise protocols, and cognitive measures, random-effects models were prespecified for all pairwise and NMA. Statistical heterogeneity was summarized using τ^2^ and I^2^, and 95% prediction intervals were reported for key comparisons where estimable.

A network plot was constructed to summarize the available direct comparisons. Statistical incoherence was assessed using the global design-by-treatment interaction model, node-splitting analyses for locally estimable comparisons, and loop-specific inconsistency factors. Treatment rankings were summarized using SUCRA values. SUCRA values were interpreted alongside effect estimates, confidence intervals, network geometry, the number of contributing trials, and certainty of evidence.

Small-study effects were explored using a comparison-adjusted funnel plot, with interpretation informed by the number of studies contributing to each comparison. A leave-one-out sensitivity analysis was conducted by sequentially excluding each study.

Exploratory subgroup analyses were conducted according to age, type of disease, duration of exercise, frequency of exercise, and exercise time. Differences between subgroups were evaluated using interaction tests. Random-effects meta-regression was performed when at least 10 studies provided sufficient covariate variation. Given the use of aggregate study-level data, these analyses were considered hypothesis-generating.

The transitivity assumption was evaluated before interpreting indirect comparisons. We compared the distribution of prespecified potential effect modifiers across treatment comparisons, including age, assessment tools, type of disease, duration of exercise, frequency of exercise, exercise time, and risk of bias. These distributions were presented in Supplementary material 12. Diagnosis-related sensitivity analyses were conducted by sequentially excluding studies of AD, PD, and MCI.

The certainty of network evidence was evaluated using the CINeMA framework. The following domains were assessed for each clinically relevant comparison: within-study bias, reporting bias, indirectness, imprecision, heterogeneity, and incoherence. Confidence in each estimate was rated as high, moderate, low, or very low. Judgments were made independently by two researchers, with disagreements resolved by consensus or consultation with a third researcher.

## 3 Results

### 3.1 Study selection

A total of 2,819 records were identified by the search, of which 1,181 were excluded prior to screening. The titles and abstracts of the remaining 1,638 records were screened. Ultimately, 123 full-text articles were assessed, and 28 eligible RCTs were included. The PRISMA flow diagram is shown in Figure 1.

**Figure 1:**
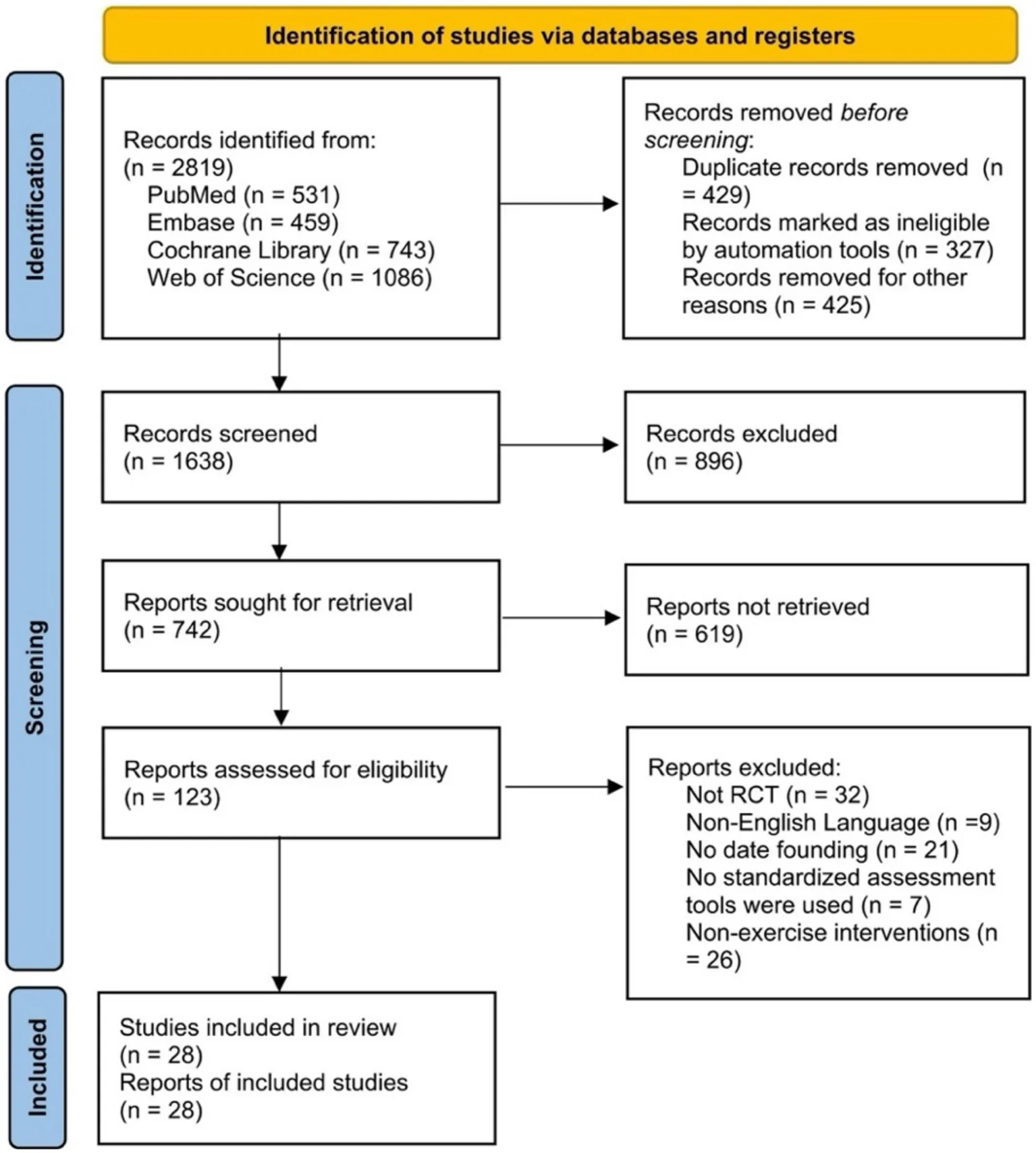
Flow chart of articles screening.

### 3.2 Characteristics of included articles

The 28 included RCTs enrolled 1,993 participants aged 58–86 years across 13 countries (China, France, America, England, Australia, Spain, Croatia, Italy, Canada, Denmark, Germany, Brazil, Netherlands). Eleven trials included participants with MCI, nine included participants with PD, and eight included participants with AD. Nineteen trials evaluated a single exercise intervention, while nine evaluated multiple exercise interventions. Fifteen trials assessed AE (walking, stepping, cycling, and running), 12 assessed MBE (Tai Chi, dance, Baduanjin, and Pilates), three assessed RT, four assessed MCE, one assessed FT, and two assessed FTr. The main assessment tools were the MoCA and MMSE. Intervention duration ranged from 2 to 32 weeks, weekly frequency ranged from 2 to 7 sessions, and exercise time ranged from 20 to 90 min. For detailed information, refer to Supplementary material 3.

### 3.3 Risk of bias assessment

According to the RoB-2 assessment, 5 articles (17.86%) were scored as high risk, 9 articles (32.14%) were judged as having some concerns, and 14 articles (50.00%) were scored as low risk. Specifically, 5 articles (17.86%) had high risk of bias in outcome measurement, 4 articles (14.29%) raised concerns regarding deviations from intended interventions, and 7 articles (25.00%) raised concerns regarding the randomization process. All articles (100%) were categorized as low risk for selection of the reported result and missing outcome data. The bias risk chart is accessible in Supplementary material 4.

### 3.4 Network meta-analysis

#### 3.4.1 Network diagram

The network evidence figure includes 7 nodes, 9 direct comparisons, 12 indirect comparisons, and 3 closed loops. The control group (CG) was the largest node, and most trials compared CG with AE or MBE, as shown in Figure 2.

**Figure 2:**
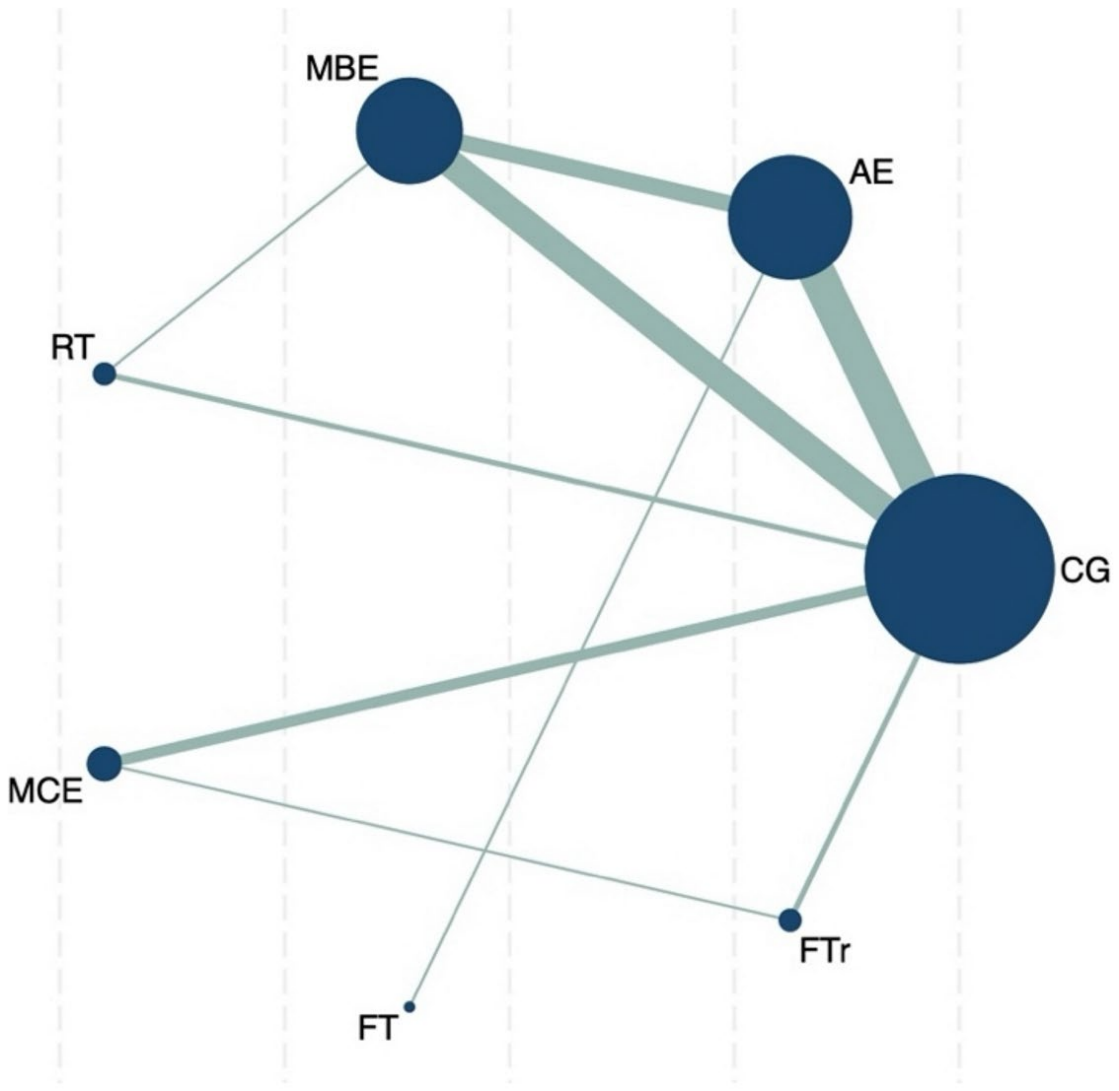
Network figure. Circles (nodes) represent the number of studies for each intervention, and lines (edges) indicate the number of direct comparisons. CG, control group; AE, aerobic exercise; MBE, mind–body exercise; RT, resistance training; MCE, multi-component exercise; FT, flexibility training; FTr, functional training.

#### 3.4.2 Results analysis

Compared with CG, AE [SMD = 0.47, 95% CI (0.18, 0.77)], MBE [SMD = 0.79, 95% CI (0.46, 1.11)], RT [SMD = 1.50, 95% CI (0.77, 2.23)], MCE [SMD = 0.83, 95% CI (0.26, 1.40)], and FTr [SMD = 0.83, 95% CI (0.13, 1.53)] showed significant benefits. There were also significant comparisons between AE and RT [SMD = −1.03, 95% CI (−1.80, −0.25)], as well as between RT and FT [SMD = 1.70, 95% CI (0.42, 2.98)]. No other between-exercise comparison was statistically significant. The results are shown in Table 2 and Supplementary materials 5, 6.

**Table 2:** Comparative analysis of various exercise interventions.

Intervention	AE	MBE	RT	MCE	FT	FTr	CG
AE	–						
MBE	−0.31(−0.68,0.05)	–					
RT	**−1.03** **(−1.80, −0.25)**	−0.71 (−1.46, 0.04)	–				
MCE	−0.36(−1.00, 0.28)	−0.05 (−0.70, 0.61)	0.67 (−0.26, 1.59)	–			
FT	0.67(−0.35, 1.70)	0.99 (−0.10, 2.07)	**1.70** **(0.42, 2.98)**	1.03 (−0.18, 2.24)	–		
FTr	−0.36(−1.11, 0.40)	−0.04 (−0.81, 0.73)	0.67 (−0.34, 1.68)	0.00 (−0.78, 0.79)	−1.03 (−2.30, 0.24)	–	
CG	**0.47** **(0.18, 0.77)**	**0.79** **(0.46, 1.11)**	**1.50** **(0.77, 2.23)**	**0.83** **(0.26, 1.40)**	−0.20 (−1.27, 0.87)	**0.83** **(0.13, 1.53)**	–

Comparisons between interventions should be interpreted from left to right. Each estimate is positioned in the cell at the junction of the column-defined and row-defined treatments. Values marked in bold confirm a significance level of *p* < 0.05. The data are outlined as standardized mean differences (SMDs) and 95% confidence intervals (CIs). AE, aerobic exercise; MBE, mind–body exercise; RT, resistance training; MCE, multi-component exercise; FT, flexibility training; FTr, functional training; CG, control group.

Based on SUCRA analysis results, RT (96.3%) had the highest probability of ranking first, followed by MCE (66.0%), FTr (64.7%), and MBE (64.3%). In addition, AE (37.8%) and FT (10.0%) had the lowest ranking probabilities, as shown in Figure 3.

**Figure 3:**
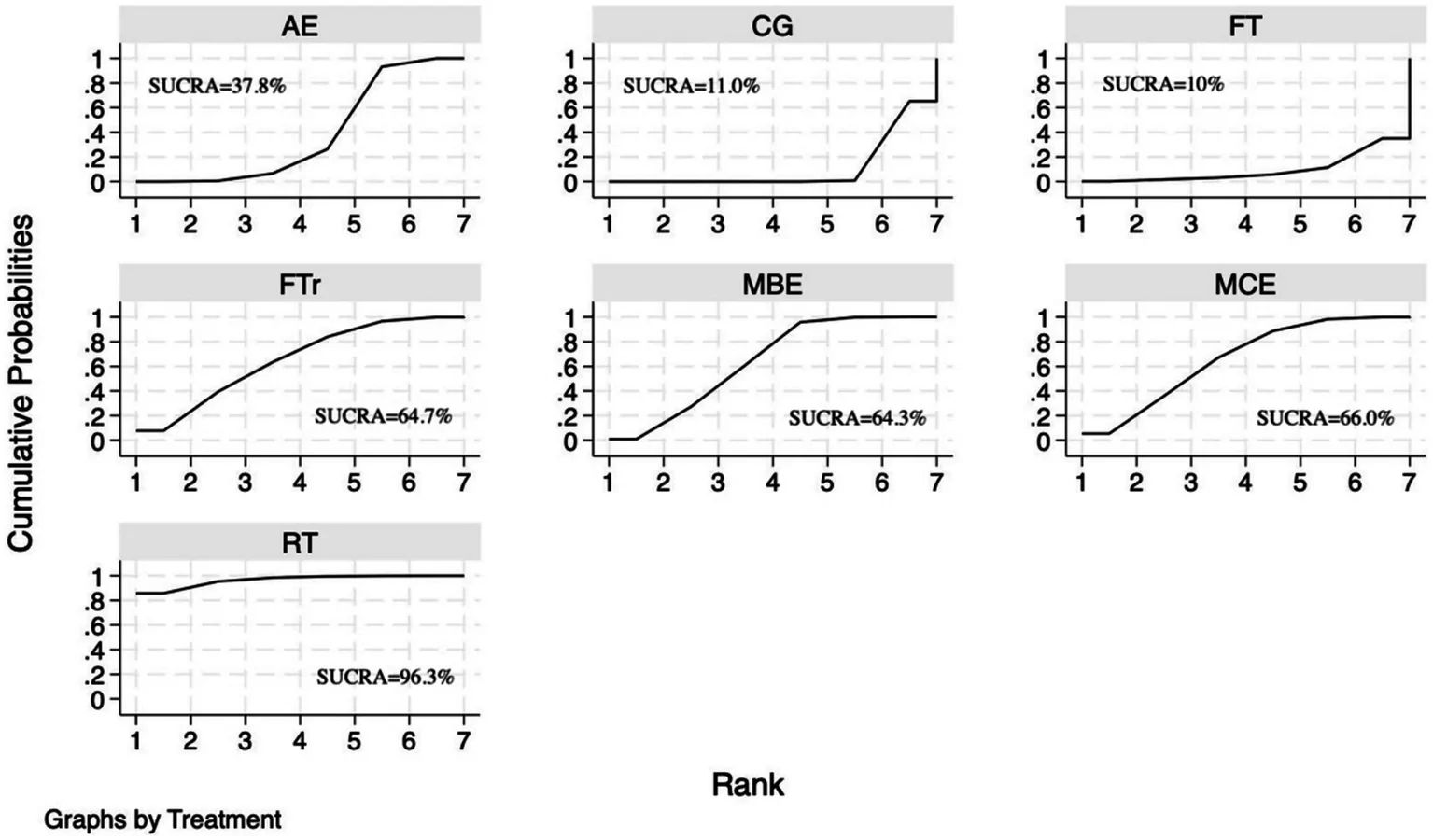
Cumulative ranking probability of various exercise interventions. The value obtained from the area under the cumulative ranking curve confirms the likelihood of each intervention being among the top-rated within the network. Higher SUCRA values indicate a greater probability that an intervention ranks among the more effective options within the network. AE, aerobic exercise; MBE, mind–body exercise; RT, resistance training; MCE, multi-component exercise; FT, flexibility training; FTr, functional training; CG, control group.

### 3.5 Heterogeneity and inconsistency analysis

The estimated between-study heterogeneity variance was τ^2^ = 0.23, and the corresponding global I^2^ was 80%, indicating substantial heterogeneity. The 95% prediction intervals for the six exercise-versus-CG comparisons were AE, −0.57 to 1.52; MBE, −0.26 to 1.84; RT, 0.24 to 2.76; MCE, −0.33 to 2.00; FT, −1.71 to 1.31; and FTr, −0.41 to 2.07. Five of the six prediction intervals included the null value, indicating that the magnitude, and in some cases the direction, of effects may vary across future settings.

The global inconsistency test was not statistically significant (χ^2^ = 0.52, df = 6, *p* = 0.998). Node-splitting and loop-specific analyses did not identify statistically significant incoherence in the three estimable loops (all *p* > 0.05). The results of the inconsistency analyses are reported in Supplementary material 7.

### 3.6 Publication bias and leave-one-out sensitivity analysis

The comparison-adjusted funnel plot showed no marked visual asymmetry, although its ability to detect small-study effects was limited by the small number of trials contributing to several comparisons. The plot is shown in Supplementary material 8.

Leave-one-out analyses showed that the pooled estimate changed little after the exclusion of any single study (Supplementary material 9).

### 3.7 Subgroup analysis and meta-regression

Exploratory subgroup analyses showed statistically significant pooled effects in studies lasting more than 12 weeks, frequencies of 3–5 sessions per week, and exercise time of 31–60 min. Interaction tests were not statistically significant for age (*p* = 0.13), type of disease (*p* = 0.24), duration of exercise (*p* = 0.60), frequency of exercise (*p* = 0.74), or exercise time (*p* = 0.26), as shown in Supplementary material 10.

Meta-regression likewise identified no statistically significant associations between these study characteristics and effect estimates (all *p* > 0.05), as shown in Supplementary material 11. These analyses therefore identified descriptive study-level patterns rather than confirmed effect modifiers.

### 3.8 Assessment of transitivity and diagnosis-based sensitivity analyses

The distributions of potential effect modifiers varied across treatment nodes, with the largest differences observed for cognitive assessment tools, type of disease, and duration of exercise (Supplementary material 12). These patterns were considered when interpreting the transitivity of the network.

RT remained the highest-ranked modality after the exclusion of AD studies [SMD = 1.15, 95% CI (0.29, 2.01)], PD studies [SMD = 2.11, 95% CI (1.26, 2.96)], or MCI studies [SMD = 1.47, 95% CI (0.50, 2.44)]. The consistency of the ranking, together with variation in the effect magnitude, suggests that the RT ranking was relatively stable but sensitive to the diagnostic composition of the network, as shown in Supplementary material 12.

### 3.9 Certainty of evidence

According to CINeMA, confidence in the evidence for exercise modalities versus control ranged from very low to low. Confidence in comparisons involving FT and FTr was generally very low because the nodes were informed by few trials and wide confidence intervals. Detailed judgments are provided in Table 3 and Supplementary material 13.

**Table 3:** Summary of comparative effects, ranking probabilities, and certainty of evidence.

Type of exercise	Number of contributing trials	SMD versus control (95% CI)	SUCRA (%)	CINeMA certainty	Principal interpretation
AE	15	0.47 (0.18, 0.77)	37.8	Low	Beneficial versus control; lower ranking probability than several alternatives
MBE	12	0.79 (0.46, 1.11)	64.3	Low	Beneficial versus control; comparatively broad evidence base
RT	3	1.50 (0.77, 2.23)	96.3	Low	Largest estimate and highest ranking probability, but based on few trials
MCE	4	0.83 (0.26, 1.40)	66.0	Low	Beneficial versus control; intervention content was heterogeneous
FT	1	−0.20 (−1.27, 0.87)	10.0	Very low	Effect remains uncertain because evidence was sparse and imprecise
FTr	2	0.83 (0.13, 1.53)	64.7	Very low	Promising estimate, but supported by very limited evidence

SUCRA indicates the relative position of each intervention within the network and should be interpreted together with effect estimates, confidence intervals, the number of contributing trials, network structure, and certainty of evidence.

## 4 Discussion

### 4.1 Principal findings

This systematic review and NMA provides a comparative overview of six exercise modalities for global cognitive function across AD, PD, and MCI. Five exercises—AE, MBE, RT, MCE, and FTr—showed beneficial effects compared with CG, indicating that cognitive improvement may be achieved through several forms of structured exercise rather than through a single intervention pathway. Among these exercises, RT produced the largest effect estimate and the highest SUCRA value, and its first-place ranking was maintained in the diagnosis-based sensitivity analyses. This consistent pattern identifies RT as a particularly promising candidate for further investigation.

Nevertheless, the ranking reflects the structure and uncertainty of the currently available evidence rather than a definitive clinical hierarchy. The RT node was informed by only three trials, substantial heterogeneity was present across the network, and no statistically significant differences were observed between RT and MBE, MCE, or FTr. The results therefore support a comparatively strong cognitive-effect signal for RT, while not establishing that it should be universally preferred over the other beneficial modalities.

The remaining interventions also provide clinically relevant evidence. MBE demonstrated a beneficial effect supported by a comparatively broad evidence base, whereas MCE and FTr produced similarly favorable estimates but were informed by fewer and more heterogeneous trials. AE yielded a smaller estimated effect but was evaluated in the largest number of studies, indicating a more established, although less highly ranked, evidence base. In contrast, the estimate for FT remained inconclusive because it was based on a single small trial with a wide confidence interval; this uncertainty should not be interpreted as evidence that FT is ineffective.

Exploratory subgroup analyses further identified statistically significant pooled effects in studies lasting more than 12 weeks, frequencies exercise 3–5 times per week, and exercise time of 31–60 min. These patterns provide descriptive support for a possible relationship between intervention dose and cognitive benefit. However, subgroup interaction tests and meta-regression did not confirm intervention duration, frequency, or exercise time as effect modifiers. The dose-related findings therefore identify potentially relevant intervention characteristics for future research rather than established prescription thresholds.

Taken together, these findings support a differentiated interpretation of exercise effects rather than a rigid ranking of modalities. RT warrants priority in future direct comparative trials, while MBE, MCE, FTr, and AE remain plausible intervention options whose clinical value may vary according to patient characteristics, therapeutic goals, feasibility, and implementation context. In addition, further dose-comparison trials are needed to determine whether specific combinations of intervention duration, frequency, and exercise time produce greater cognitive benefits.

### 4.2 Potential mechanisms

Several biological and behavioral pathways may contribute to the cognitive benefits observed across exercise modalities. AE may enhance cerebral perfusion, cardiorespiratory fitness, and neurotrophic signaling, thereby supporting neuronal metabolism and synaptic plasticity (20, 49, 50). RT may influence cognition through muscle-brain signaling pathways involving insulin-like growth factor-1 (IGF-1), brain-derived neurotrophic factor (BDNF), and other exercise-induced myokines (49, 58). By contrast, MBE and FTr may place greater demands on attention, balance, coordination, motor planning, and cognitive-motor integration (22, 23, 27).

The beneficial estimates across several exercise categories suggest that cognitive improvement may arise through multiple, partly overlapping pathways rather than a single modality-specific mechanism. Physical adaptations, cognitive engagement, social interaction, and repeated participation in structured activity may jointly contribute to the observed effects. Because the present NMA synthesized global cognitive outcomes rather than biomarkers or neuroimaging measures, these mechanisms remain biologically plausible explanations rather than pathways demonstrated directly by the current findings. Trials integrating cognitive assessments with circulating biomarkers, neuroimaging, and physical-function measures are needed to clarify whether different exercise modalities act through distinct or shared mechanisms.

### 4.3 Interpretation of other exercise modalities

The present findings indicate that exercise modalities may offer different combinations of cognitive effects, physical benefits, and implementation advantages. MBE produced a favorable effect estimate and was supported by a comparatively broad evidence base. Modalities such as Tai Chi, dance, Baduanjin, and Pilates combine movement with attentional control, balance, rhythm, and coordination, which may be particularly relevant for individuals who benefit from lower-impact and cognitively engaging activities (22, 23).

MCE also showed a beneficial effect and may be useful when treatment goals extend beyond cognition to include strength, balance, mobility, endurance, and flexibility (26). The MCE node nevertheless encompassed programs with substantially different combinations and emphases. Its pooled estimate should therefore be understood as the effect of a broad multicomponent approach rather than evidence for a specific optimal combination. Factorial or dismantling trials could help identify which components contribute most strongly to cognitive improvement.

FTr showed a similarly favorable estimate, suggesting potential value for interventions that incorporate task-oriented movements and activities related to everyday function. Its evidence base, however, consisted of only two trials with heterogeneous protocols, making this finding promising but still preliminary.

AE had a smaller estimated cognitive effect than several higher-ranked modalities but was evaluated in the largest number of studies. Its comparatively extensive evidence base, broad feasibility, and additional cardiorespiratory and metabolic benefits remain clinically important (51, 52). The lower SUCRA value of AE should therefore not be interpreted as evidence of limited clinical usefulness, particularly because the present review assessed global cognition rather than the full range of health outcomes associated with AE.

### 4.4 Exploratory associations with intervention dose

Studies lasting more than 12 weeks, frequencies exercise 3–5 times per week, and exercise time of 31–60 min showed statistically significant pooled effects within their respective subgroups. These patterns identify intervention characteristics that may deserve priority in future dose-comparison research. They are also broadly consistent with the possibility that repeated and sustained exposure is needed for exercise-related physiological and cognitive adaptations to develop (53, 54).

Formal subgroup interaction tests and Meta-regression did not, however, confirm duration, frequency, or exercise time as treatment-effect modifiers. This distinction is important because a significant effect within one subgroup does not demonstrate that it differs from the effect observed in another subgroup. In addition, exercise duration, frequency, exercise time, modality, diagnosis, and control design were correlated across studies, making it difficult to isolate an independent dose–response relationship from aggregate data.

The observed dose patterns should consequently be regarded as hypotheses for future trials rather than fixed therapeutic thresholds. Direct comparisons of different exercise doses within the same modality and diagnostic population would provide a more reliable basis for determining whether longer interventions or particular frequency and session-duration ranges produce greater cognitive benefits.

### 4.5 FT and CG design

FT had the lowest ranking probability and was not statistically distinguishable from CG. Because this estimate was informed by only one small trial and had a wide confidence interval, the result primarily reflects uncertainty rather than convincing evidence of no cognitive effect.

The interpretation of FT is also relevant to the design of exercise trials. Stretching and low-intensity flexibility programs may serve as active control conditions when researchers seek to balance participant contact, expectations, social interaction, and engagement in structured activity (55, 56). Such controls may improve methodological comparability, but they are not necessarily physiologically or psychologically inert (56, 57). Researchers should therefore describe their intensity, content, credibility, and anticipated effects in sufficient detail. Three-arm designs incorporating an experimental exercise intervention, an active exercise control, and a usual-care or no-exercise group may better distinguish modality-specific effects from the nonspecific effects of participation and attention (56).

### 4.6 Transitivity, heterogeneity, and certainty of evidence

Combining evidence from AD, PD, and MCI created a common framework for comparing exercise modalities across bodies of literature that have usually developed separately. This approach broadens the comparative evidence base and allows potentially shared exercise effects on global cognition to be examined. Its interpretation nevertheless depends on whether important effect modifiers are sufficiently comparable across treatment comparisons.

The distributions of cognitive assessment tools, diagnostic categories, and intervention duration differed across exercise nodes, with additional variation in age, weekly frequency, session duration, and risk of bias. Moreover, important clinical characteristics, including disease severity, baseline cognitive status, and medication use, were incompletely reported and could not be compared adequately across treatment nodes. Motor impairment, exercise intensity, and adherence were also not consistently available. These measured and unmeasured differences may have affected the transitivity of some indirect comparisons and should be considered when interpreting the network estimates. Diagnosis-based sensitivity analyses provided some reassurance: RT remained the highest-ranked modality after the sequential exclusion of AD, PD, and MCI studies. The corresponding RT effect estimate varied from 1.15 to 2.11, indicating that the direction and ranking signal were relatively stable, although the magnitude remained sensitive to network composition.

Overall statistical heterogeneity remained substantial, and five of the six exercise-versus-control prediction intervals included the null value. This suggests that effects may differ across future studies and clinical settings. Likely contributors included diagnostic diversity, variation in cognitive tools, differences in exercise intensity and dose, control design, and risk of bias. This heterogeneity reduces confidence that a single pooled estimate or ranking will apply uniformly across clinical settings.

CINeMA ratings ranged from low to very low, mainly because of within-study bias, reporting bias, imprecision, and heterogeneity. These ratings do not negate the observed beneficial estimates; rather, they define the level of confidence that can be placed in their magnitude and ranking. The network results are therefore most appropriately interpreted as comparative signals within the current evidence base, not as evidence of uniform effects across all three diagnostic populations.

### 4.7 Limitations

Several limitations should be considered. The evidence network was uneven, with RT, FTr, and FT informed by only three, two, and one trials, respectively. Estimates and rankings for these nodes were consequently more sensitive to individual studies than those supported by broader evidence bases.

Clinical and methodological diversity was also considerable. Included studies differed in diagnosis, exercise content and dose, cognitive instruments, and control conditions. Although transitivity and incoherence were assessed, incompletely reported factors such as disease severity, baseline cognitive status, medication use, motor impairment, exercise intensity, and adherence could not be fully evaluated.

Subgroup analyses and Meta-regression were based on aggregate study-level data and relatively few studies, leaving them vulnerable to ecological bias, residual confounding, and limited statistical power. In addition, the review focused on global cognition and could not determine whether exercise modalities differed in their effects on specific domains such as memory, executive function, or attention. The review did not synthesize safety, adherence, or adverse-event outcomes, limiting comparisons of acceptability and clinical feasibility.

### 4.8 Clinical implications and future research

The findings provide a comparative framework for considering exercise interventions according to patient characteristics, therapeutic objectives, and implementation conditions. They support the potential cognitive value of several structured exercise modalities rather than a single universally preferred approach.

RT showed the largest effect estimate and the most favorable ranking; however, this finding was based on only three trials and low-certainty evidence. RT should therefore be regarded as a promising candidate for further investigation rather than a preferred intervention for routine clinical use. It may be considered when improving muscle strength and physical function is also a treatment goal, provided that supervision needs, frailty, fall risk, and the individual’s capacity to perform progressive loading safely are carefully assessed. MBE may be suitable for individuals requiring lower-impact, minimally equipped, and cognitively engaging programs. MCE may be particularly relevant when cognition, mobility, balance, and strength are simultaneous treatment targets. AE remains widely accessible and offers cardiovascular and general health benefits in addition to its observed cognitive effect. Evidence for FTr is encouraging but requires further replication.

Exercise selection should therefore be individualized according to diagnosis, disease severity, baseline cognitive and motor status, comorbidities, medication use, accessibility, patient preference, and available supervision. The present findings may help identify priorities for future research, but the current evidence does not support a definitive clinical hierarchy among exercise modalities.

Future research should prioritize adequately powered head-to-head trials within clearly defined diagnostic populations. Exercise protocols should report modality, intensity, progression, frequency, session length, adherence, and adverse events using established reporting standards. Direct dose-comparison trials are needed before specific exercise thresholds can be recommended. Studies integrating cognitive subdomains, physical-function outcomes, biomarkers, and neuroimaging may clarify why particular patients respond differently to different forms of exercise. Individual participant data NMA may further help identify diagnosis-specific and patient-level effect modifiers.

## 5 Conclusion

In this exploratory NMA, AE, MBE, RT, MCE, and FTr were associated with improvements in global cognitive function compared with CG. RT showed the largest effect estimate and the highest ranking probability, identifying it as a promising modality for further direct comparative research. MBE, MCE, FTr, and AE also demonstrated potentially meaningful benefits, although the amount and certainty of evidence varied across modalities. Exploratory analyses further suggested larger pooled effects in studies lasting more than 12 weeks, delivered 3–5 times per week, and using sessions of 31–60 min. Because these dose-related patterns were not confirmed by formal subgroup interaction tests or meta-regression, they should be regarded as hypotheses for future trials rather than established prescription thresholds. Given the substantial heterogeneity, sparse evidence for several modalities, and low to very low certainty, the rankings are best interpreted as comparative signals rather than a definitive cross-diagnostic hierarchy. Overall, the findings support individualized exercise selection according to diagnosis, physical capacity, therapeutic goals, feasibility, and patient preference, while highlighting the need for adequately powered head-to-head and dose-comparison trials.

## Data Availability

The original contributions presented in the study are included in the article/Supplementary material, further inquiries can be directed to the corresponding author.
