# Supplementary material for: Comparative effects of six exercise modalities on global cognitive function in Alzheimer’s disease, Parkinson’s disease, and mild cognitive impairment: a systematic review and network meta-analysis
Source: Front Neurol. 2026 Sep 25;17:1877537. doi: 10.3389/fneur.2026.1877537 (PMC13649996; doi:10.3389/fneur.2026.1877537)
Supplement: Supplementary file 1 [file Supplementary_file_1.docx]

**Supplementary Material**

**Index of supplementary material**

[Supplementary 1 PRISMA NMA checklist	2](#_Toc1715498207)

[Supplementary 2 Search strategy	6](#_Toc262920427)

[Supplementary 3 Characteristics of included studies	8](#_Toc1529754710)

[Supplementary 4 Cochrane risk-of-bias assessment	11](#_Toc913189086)

[Supplementary 5 Pairwise comparison	12](#_Toc2050826940)

[Supplementary 6 Comparison of direct and indirect estimates	12](#_Toc1135846230)

[Supplementary 7 Heterogeneity and inconsistency analyses 	13](#_Toc1185449427)

[Supplementary 8 Publication bias	14](#_Toc1642726370)

[Supplementary 9 Sensitivity analyses	14](#_Toc1252334758)

[Supplementary 10 Subgroup analyses	15](#_Toc503053459)

[Supplementary 11 Meta-regression	15](#_Toc176367174)

[Supplementary 12 Transitivity assessment	15](#_Toc675660558)

[Supplementary 13 CINeMA certainty-of-evidence assessment	17](#_Toc2080956617)

# Supplementary 1 PRISMA NMA checklist

| **Section/Topic** | **Item #** | **Checklist Item** | **Section headings** |
| --- | --- | --- | --- |
| **TITLE** |  |  |  |
| Title | 1 | Identify the report as a systematic review *incorporating a network meta-analysis (or related form of meta-analysis).* | Title |
|  |  |  |  |
| **ABSTRACT** |  |  |  |
| Structured summary | 2 | Provide a structured summary including, as applicable:  **Background:** main objectives  **Methods:** data sources; study eligibility criteria, participants, and interventions; study appraisal; and *synthesis methods, such as network meta-analysis.*  **Results:** number of studies and participants identified; summary estimates with corresponding confidence/credible intervals; *treatment rankings may also be discussed. Authors may choose to summarize pairwise comparisons against a chosen treatment included in their analyses for brevity.*  **Discussion/Conclusions:** limitations; conclusions and implications of findings.  **Other:** primary source of funding; systematic review registration number with registry name. | Abstract |
|  |  |  |  |
| **INTRODUCTION** |  |  |  |
| Rationale | 3 | Describe the rationale for the review in the context of what is already known*, including mention of why a network meta-analysis has been conducted.* | 1 Introduction |
| Objectives | 4 | Provide an explicit statement of questions being addressed, with reference to participants, interventions, comparisons, outcomes, and study design (PICOS). | 1 Introduction |
|  |  |  |  |
| **METHODS** |  |  |  |
| Protocol and registration | 5 | Indicate whether a review protocol exists and if and where it can be accessed (e.g., Web address); and, if available, provide registration information, including registration number. | 2.1 Protocol and registration |
| Eligibility criteria | 6 | Specify study characteristics (e.g., PICOS, length of follow-up) and report characteristics (e.g., years considered, language, publication status) used as criteria for eligibility, giving rationale. *Clearly describe eligible treatments included in the treatment network, and note whether any have been clustered or merged into the same node (with justification).* | 2.3 Eligibility criteria |
| Information sources | 7 | Describe all information sources (e.g., databases with dates of coverage, contact with study authors to identify additional studies) in the search and date last searched. | 2.2 Data sources and search approaches |
| Search | 8 | Present full electronic search strategy for at least one database, including any limits used, such that it could be repeated. | Supplementary 2 |
| Study selection | 9 | State the process for selecting studies (i.e., screening, eligibility, included in systematic review, and, if applicable, included in the meta-analysis). | 2.5 Data selection and extraction |
| Data collection process | 10 | Describe method of data extraction from reports (e.g., piloted forms, independently, in duplicate) and any processes for obtaining and confirming data from investigators. | 2.5 Data selection and extraction |
| Data items | 11 | List and define all variables for which data were sought (e.g., PICOS, funding sources) and any assumptions and simplifications made. | 2.3 Eligibility criteria |
| **Geometry of the network** | **S1** | Describe methods used to explore the geometry of the treatment network under study and potential biases related to it. This should include how the evidence base has been graphically summarized for presentation, and what characteristics were compiled and used to describe the evidence base to readers. | 3.4.1 Network diagram |
| Risk of bias within individual studies | 12 | Describe methods used for assessing risk of bias of individual studies (including specification of whether this was done at the study or outcome level), and how this information is to be used in any data synthesis. | 2.6 Risk of bias assessment |
| Summary measures | 13 | State the principal summary measures (e.g., risk ratio, difference in means). *Also describe the use of additional summary measures assessed, such as treatment rankings and surface under the cumulative ranking curve (SUCRA) values, as well as modified approaches used to present summary findings from meta-analyses.* | 2.7 Statistical analysis |
| Planned methods of analysis | 14 | Describe the methods of handling data and combining results of studies for each network meta-analysis. This should include, but not be limited to:   - *Handling of multi-arm trials;* - *Selection of variance structure;* - *Selection of prior distributions in Bayesian analyses; and* - *Assessment of model fit.* | 2.7 Statistical analysis |
| **Assessment of Inconsistency** | **S2** | Describe the statistical methods used to evaluate the agreement of direct and indirect evidence in the treatment network(s) studied. Describe efforts taken to address its presence when found. | 2.7 Statistical analysis; Supplementary 7 |
| Risk of bias across studies | 15 | Specify any assessment of risk of bias that may affect the cumulative evidence (e.g., publication bias, selective reporting within studies). | 3.8 Assessment of transitivity and diagnosis-based sensitivity analyses; 3.9 Certainty of evidence;  Supplementary 4 |
| Additional analyses | 16 | Describe methods of additional analyses if done, indicating which were pre-specified. This may include, but not be limited to, the following:   - Sensitivity or subgroup analyses; - Meta-regression analyses; - *Alternative formulations of the treatment network; and* - *Use of alternative prior distributions for Bayesian analyses (if applicable).* | 3.7 Subgroup analysis and meta-regression  ; Supplementary 9;  Supplementary 10 |
|  |  |  |  |
| **RESULTS†** |  |  |  |
| Study selection | 17 | Give numbers of studies screened, assessed for eligibility, and included in the review, with reasons for exclusions at each stage, ideally with a flow diagram. | 3.1 Study selection |
| **Presentation of network structure** | **S3** | Provide a network graph of the included studies to enable visualization of the geometry of the treatment network. | Figure 2 |
| **Summary of network geometry** | **S4** | Provide a brief overview of characteristics of the treatment network. This may include commentary on the abundance of trials and randomized patients for the different interventions and pairwise comparisons in the network, gaps of evidence in the treatment network, and potential biases reflected by the network structure. | 3.4.1 Network diagram; Supplementary 5; Supplementary 6 |
| Study characteristics | 18 | For each study, present characteristics for which data were extracted (e.g., study size, PICOS, follow-up period) and provide the citations. | 3.2 Characteristics of included articles; Supplementary 3 |
| Risk of bias within studies | 19 | Present data on risk of bias of each study and, if available, any outcome level assessment. | 3.6 Publication bias and leave-one-out sensitivity analysis; Supplementary 4 |
| Results of individual studies | 20 | For all outcomes considered (benefits or harms), present, for each study: 1) simple summary data for each intervention group, and 2) effect estimates and confidence intervals. *Modified approaches may be needed to deal with information from larger networks.* | Supplementary 4-10 |
| Synthesis of results | 21 | Present results of each meta-analysis done, including confidence/credible intervals. *In larger networks, authors may focus on comparisons versus a particular comparator (e.g. placebo or standard care), with full findings presented in an appendix. League tables and forest plots may be considered to summarize pairwise comparisons.* If additional summary measures were explored (such as treatment rankings), these should also be presented. | Supplementary 5;  Supplementary 6 |
| **Exploration for inconsistency** | **S5** | Describe results from investigations of inconsistency. This may include such information as measures of model fit to compare consistency and inconsistency models, *P* values from statistical tests, or summary of inconsistency estimates from different parts of the treatment network. | 3.5 Heterogeneity and inconsistency analysis; Supplementary 7 |
| Risk of bias across studies | 22 | Present results of any assessment of risk of bias across studies for the evidence base being studied. | 3.6 Publication bias and leave-one-out sensitivity analysis; Supplementary 4 |
| Results of additional analyses | 23 | Give results of additional analyses, if done (e.g., sensitivity or subgroup analyses, meta-regression analyses*, alternative network geometries studied, alternative choice of prior distributions for Bayesian analyses,* and so forth). | 3.7 Subgroup analysis and meta-regressio; Supplementary 9;  Supplementary 10 |
|  |  |  |  |
| **DISCUSSION** |  |  |  |
| Summary of evidence | 24 | Summarize the main findings, including the strength of evidence for each main outcome; consider their relevance to key groups (e.g., healthcare providers, users, and policy-makers). | 4.1 Principal findings |
| Limitations | 25 | Discuss limitations at study and outcome level (e.g., risk of bias), and at review level (e.g., incomplete retrieval of identified research, reporting bias). *Comment on the validity of the assumptions, such as transitivity and consistency. Comment on any concerns regarding network geometry (e.g., avoidance of certain comparisons).* | 4.7 Limitations |
| Conclusions | 26 | Provide a general interpretation of the results in the context of other evidence, and implications for future research. | 5 Conclusions |
|  |  |  |  |
| **FUNDING** |  |  |  |
| Funding | 27 | Describe sources of funding for the systematic review and other support (e.g., supply of data); role of funders for the systematic review. This should also include information regarding whether funding has been received from manufacturers of treatments in the network and/or whether some of the authors are content experts with professional conflicts of interest that could affect use of treatments in the network. | - |

PICOS = population, intervention, comparators, outcomes, study design.

* Text in italics indicateS wording specific to reporting of network meta-analyses that has been added to guidance from the PRISMA statement.

† Authors may wish to plan for use of appendices to present all relevant information in full detail for items in this section.

# Supplementary 2 Search strategy

Table 1. Search strategy in PubMed

| Step | Search strategy | Results | Date/Time |
| --- | --- | --- | --- |
| #14 | (((((((((((("Exercise"[Mesh]) OR (Physical Exercise[Title/Abstract])) OR (Physical Activity[Title/Abstract])) OR (Exercise Training[Title/Abstract])) OR (Exercise Therapy[Title/Abstract])) OR (Motor Activity[Title/Abstract])) OR (Aerobic Exercise[Title/Abstract])) OR (Resistance Training[Title/Abstract])) OR (Strength Training[Title/Abstract])) OR (Isometric Exercises[Title/Abstract])) AND (((((("Cognition"[Mesh]) OR (Cognitive Function[Title/Abstract])) OR (Cognitions[Title/Abstract])) OR (Function, Cognitive[Title/Abstract])) OR (Cognitive Functions[Title/Abstract])) OR (Functions, Cognitive[Title/Abstract]))) AND ((((("Parkinson Disease"[Mesh]) OR (Parkinson's Disease[Title/Abstract])) OR (PD[Title/Abstract])) OR ((("Alzheimer Disease"[Mesh]) OR (Alzheimer's Diseases[Title/Abstract])) OR (AD[Title/Abstract]))) OR ((("Cognitive Dysfunction"[Mesh]) OR (Cognitive Impairment, Mild[Title/Abstract])) OR (MCI[Title/Abstract])))) AND (("Randomized Controlled Trials as Topic"[Mesh]) OR (Randomized Controlled Trial[Publication Type])) | 531 | 2026/1/26  2:03:28 |
| #13 | ("Randomized Controlled Trials as Topic"[Mesh]) OR (Randomized Controlled Trial[Publication Type]) | 841,735 | 2026/1/26  2:00:11 |
| #12 | "Randomized Controlled Trials as Topic"[Mesh] | 196,403 | 2026/1/26  1:59:40 |
| #11 | (((("Parkinson Disease"[Mesh]) OR (Parkinson's Disease[Title/Abstract])) OR (PD[Title/Abstract])) OR ((("Alzheimer Disease"[Mesh]) OR (Alzheimer's Diseases[Title/Abstract])) OR (AD[Title/Abstract]))) OR ((("Cognitive Dysfunction"[Mesh]) OR (Cognitive Impairment, Mild[Title/Abstract])) OR (MCI[Title/Abstract])) | 634,550 | 2026/1/26  1:59:15 |
| #10 | (("Cognitive Dysfunction"[Mesh]) OR (Mild Cognitive Impairment[Title/Abstract])) OR (MCI[Title/Abstract]) | 70,341 | 2026/1/26  1:55:12 |
| #9 | "Cognitive Dysfunction"[Mesh] | 52,652 | 2026/1/26  1:54:42 |
| #8 | (("Alzheimer Disease"[Mesh]) OR (Alzheimer's Diseases[Title/Abstract])) OR (AD[Title/Abstract]) | 279,776 | 2026/1/26  1:54:19 |
| #7 | "Alzheimer Disease"[Mesh] | 138,785 | 2026/1/26  1:53:55 |
| #6 | (("Parkinson Disease"[Mesh]) OR (Parkinson's Disease[Title/Abstract])) OR (PD[Title/Abstract]) | 319,055 | 2026/1/26  1:53:17 |
| #5 | "Parkinson Disease"[Mesh] | 93,334 | 2026/1/26  1:52:44 |
| #4 | ((((("Cognition"[Mesh]) OR (Cognitive Function[Title/Abstract])) OR (Cognitions[Title/Abstract])) OR (Function, Cognitive[Title/Abstract])) OR (Cognitive Functions[Title/Abstract])) OR (Functions, Cognitive[Title/Abstract]) | 292,356 | 2026/1/26  1:51:55 |
| #3 | "Cognition"[Mesh] | 224,832 | 2026/1/26  1:47:28 |
| #2 | ((((((((("Exercise"[Mesh]) OR (Physical Exercise[Title/Abstract])) OR (Physical Activity[Title/Abstract])) OR (Exercise Training[Title/Abstract])) OR (Exercise Therapy[Title/Abstract])) OR (Motor Activity[Title/Abstract])) OR (Aerobic Exercise[Title/Abstract])) OR (Resistance Training[Title/Abstract])) OR (Strength Training[Title/Abstract])) OR (Isometric Exercises[Title/Abstract]) | 442,176 | 2026/1/26  1:46:50 |
| #1 | "Exercise"[Mesh] | 282,103 | 2026/1/26  1:44:51 |

# Supplementary 3 Characteristics of included studies

Table 2. Core study and intervention characteristics

| Study | Country | Type of Disease | Sample Size (EG/CG) | Age (M±SD) | Duration/Frequency/Time | Intervention methods | Assessment tools | Type of exercise |
| --- | --- | --- | --- | --- | --- | --- | --- | --- |
| Chen et al., (2023) | China | MCI | 107/110/111 | 67.56±4.99/ 67.46±4.73/ 67.62±5.35 | 24 weeks; 3 times/Week; 60 min/time | EG_1_: 24-Form Tai Chi; EG_2_: Fitness Walking. | MoCA | MBE/ AE |
| Zhu et al., (2018) | China | MCI | 27/27 | 70.3 ±6.7/ 69.0 ±7.3 | 12 weeks; 3 times/Week; 35min/time | EG: Dance | MoCA | MBE |
| Song & Yu, (2019) | China | MCI | 60/60 | 76.22 ± 5.76/ 75.33 ± 6.78 | 16 weeks; 3 times/week; 60 min/time | EG: Step Exercise Program | MoCA | AE |
| Wei & Ji, (2014) | China | MCI | 30/30 | 66.73 ± 5.48/ 65.27 ± 4.63 | 24 weeks; 5 times/week; 30 min/time | EG: FTr | MMSE | FTr |
| Chang et al., (2024) | China | PD | 14/16/13 | 64.43±7.37/ 66.31±6.54/ 63.15±7.95 | EG_1_: 12 weeks; 3 times/week; 30 min/time EG_2_: 12 weeks; 2 times/week; 60 min/time | EG_1_: Stationary Bike Training EG_2_: Yang Style 24-Form Tai Chi | MoCA | AE/ MBE |
| Ayari et al., (2023) | France | MCI | 12/11 | 79.8 ± 7.7/ 77.2 ± 5.3 | 16 weeks; 60 min/week | EG: Improvisational dance  CG: AE training | MMSE | MBE/ AE |
| Yu et al., (2021) | America | AD | 32/64 | 77.4±6.6/ 77.5±7.1 | 24 weeks; 3 times/ week; 20-50 min/time | EG: Cycling  CG: Stretching exercises | ADAS-Cog | AE/FT |
| Tao et al., (2019) | China | MCI | 20/17/20 | 66.17±4.17/ 64.32±2.60/ 65.97±5.66 | 24 weeks; 3 times/week; 60 min/time | EG_1_: Baduan Jin EG_2_: Brisk walking | MoCA | MBE/ AE |
| Silva-Batista et al., (2016) | England | PD | 17/15 | 64.6 ± 10.5/ 66.8 ± 8.9 | 12 weeks; 3 times/week; 80-90 min/time | EG: Perform free weight training for the upper and lower limbs utilizing equipment. | MoCA | RT |
| Lautenschlager et al., (2008) | Australian | AD | 85/85 | 68.6±8.7/ 68.7±8.5 | 24 weeks; 3 times/week; 50 min/time | EG: Walking, strength training, aerobic training | ADAS-Cog | MCE |
| Liu et al., (2022) | China | MCI | 17/17 | 73.2 ± 6.3/ 73.4 ± 6.5 | 12 weeks; 3 times/week; 50 min/time | EG: Tai Chi | MoCA | MBE |
| Lin et al., (2025) | China | MCI | 18/18/18 | 86.17 ± 6.02/ 85.28 ± 4.65/  81.96 ± 6.12 | 12 weeks; 3 times/week; 30 min/time | EG_1_: Tai Chi  EG_2_: Walking on level ground | MoCA | MBE/ AE |
| Sánchez-Alcalá et al., (2025) | Spain | MCI | 47/45 | 71.43 ± 2.97/  72.24 ± 2.92 | 12 weeks; 2 times/week; 60 min/time | EG: Various dance styles | MoCA | MBE |
| Yang et al., (2015) | China | AD | 25/25 | 72.00±6.69/ 71.92±7.28 | 12 weeks; 3 times/week; 40 min/time | EG: Cycling | MMSE | AE |
| Greblo Jurakic et al., (2017) | Croatia | MCI | 14/14 | 70.4±3.93/ 72.1±4.42/ | EG: 8 weeks; 3 times/week; 30 min/time CG: 8 weeks; 3 times/week; 60 min/time | EG: Pilates CG: RT | MoCA | MBE/ RT |
| Picelli et al., (2016) | Italy | PD | 9/8 | 71.2 ±9.2/ 71.6±7.2 | 4 weeks; 3 times/week; 45 min/time | EG: Treadmill Training | MoCA | AE |
| Nadeau et al., (2014) | Canada | PD | 11/11 | 64.0±6.6/ 64.3±5.6 | 24 weeks; 3 times/week; 60 min/time | EG: Treadmill Training | MMSE | AE |
| McKee & Hackney, (2013) | America | PD | 23/8 | 68.4±7.5/ 74.4±6.5 | 12 weeks; 2 times/week; 90 min/time | EG: Tango dance | MoCA | MBE |
| Hoffmann et al., (2016) | Denmark | AD | 102/88 | 69.8±7.4/ 71.3±7.3 | 16 weeks; 3 times/week; 60 min/time | EG: Cycling, treadmill workouts | MMSE | AE |
| Holthoff et al., (2015) | Germany | AD | 13/14 | 72.4±4.34/ 70.67±5.41 | 12 weeks; 3 times/week; 30 min/time | EG: RT | MMSE | RT |
| Vreugdenhil et al., (2012) | Australian | AD | 20/20 | 73.5±7.9/ 74.7±8.2 | 16 weeks; 7 times/week; 30 min/time | EG: Brisk walking | MMSE | AE |
| Fajersztajn et al., (2008) | Brazil | AD | 5/5 | 78.40±6.43/ 76.40±7.50 | 12 weeks; 60 min/week | EG: Balance training, stretching exercises (walking, ball exercises, FTr) | MMSE | MCE |
| de Oliveira Silva et al., (2019) | Brazil | AD | 12/7 | 71.85±5.69/ 78.20 ± 5.26 | 12 weeks; 2 times/week; 60 min/time | EG: Aerobic training, strength training, balance training, stretching training | MMSE | MCE |
| Tsai et al., (2025) | China | PD | 21/20/20 | 64.67 ± 8.05/ 65.05 ± 6.80/ 67.00 ± 7.07 | EG_1_: 12 weeks; 2 times/week; 60 min/time EG_2_: 12 weeks; 3 times/week; 30 min/time | EG_1_: Yang Style Tai Chi Chuan (Short Form).  EG_2_: Stationary bicycle | MMSE | MBE/ AE |
| Kolk et al., (2019) | Netherlands | PD | 65/65 | 59.3±8.3/ 59.4±9.3 | 24 weeks; 3 times/week; 30-45 min/time | EG: Cycling | MoCA | AE |
| Rios Romenets et al., (2015) | Canada | PD | 18/15 | 63.2±9.9/ 64.3±8.1 | 12 weeks; 2 times/week; 60 min/time | EG: Tango dance | MoCA | MBE |
| Gobbi et al., (2021) | Brazil | PD | 57/48/47 | 69.6 ±8.2/  67.8 ±9.1/  69.5 ±7.6 | 32 weeks; 2 times/week; 60 min/time | EG_1_: AE, RT; EG_2_: FTr: balance training, strength training | MMSE | MCE/ FTr |
| Huang et al., (2024) | China | MCI | 10/10 | 63.80 ± 7.50/  58.30 ± 8.10 | 2 weeks; 5 times/week; 40 min/time | EG: Treadmill Training | MoCA | AE |

# Supplementary 4 Cochrane risk-of-bias assessment

Figure 1. Risk of Bias Graph

Figure 2. Risk of Bias Summary

# Supplementary 5 Pairwise comparison

Figure 3. Pairwise comparison forest figure

Note: AE: aerobic exercise; MBE: mind–body exercise; RT: resistance training; MCE: multi-component exercise; FT: flexibility training; FTr: functional training; CG: control group.

# Supplementary 6 Comparison of direct and indirect estimates

Figure 4. Comparison of contributions between direct and indirect estimates

Note: A: control group; B: aerobic exercise; C: mind-body exercise; D: resistance training; E: multi-component exercise; F: flexibility training; G: functional training.

# Supplementary 7 Heterogeneity and inconsistency analyses

Table 3. Estimated global inconsistency

| Network outcome | Chi square | Prob > Chi2 |
| --- | --- | --- |
| cognitive function | Chi2 (6) = 0.52 | Prob > Chi2 = 0.998 |

Table4. Node-splitting

| Side | Direct | | Indirect | | Difference | | *P* > z | tau |
| --- | --- | --- | --- | --- | --- | --- | --- | --- |
|  | Coef. | Std. Err. | Coef. | Std. Err. | Coef. | Std. Err. |  |  |
| CG-AE | 0.500 | 0.160 | 0.180 | 0.540 | 0.320 | 0.564 | 0.570 | 0.483 |
| CG-MBE | 0.800 | 0.184 | 0.732 | 0.418 | 0.067 | 0.457 | 0.883 | 0.485 |
| CG-RT | 1.444 | 0.463 | 1.618 | 0.648 | -0.173 | 0.800 | 0.828 | 0.483 |
| CG-MCE* | 0.846 | 0.303 | 0.507 | 1.439 | 0.339 | 1.472 | 0.818 | 0.487 |
| CG-FTr* | 0.785 | 0.382 | 1.232 | 1.168 | -0.447 | 1.230 | 0.716 | 0.484 |
| AE-MBE | 0.343 | 0.238 | 0.261 | 0.321 | 0.081 | 0.400 | 0.839 | 0.487 |
| AE-FT* | -0.673 | 0.522 | -1.171 | 63.240 | 0.498 | 63.242 | 0.994 | 0.473 |
| MBE-RT | 0.822 | 0.625 | 0.648 | 0.493 | 0.174 | 0.796 | 0.827 | 0.483 |
| MCE FTr | 0.121 | 0.522 | -0.193 | 0.648 | 0.314 | 0.832 | 0.706 | 0.484 |

Note: AE: aerobic exercise; MBE: mind–body exercise; RT: resistance training; MCE: multi-component exercise; FT: flexibility training; FTr: functional training; CG: control group.

Table 5. Loop-specific heterogeneity estimates

| Loop | IF | seIF | Z | *P* | 95%CI | Loop-Heterog tau^2^ |
| --- | --- | --- | --- | --- | --- | --- |
| CG-MCE-FTr | 0.341 | 0.506 | 0.674 | 0.500 | (0.00, 1.33) | 0.071 |
| CG-MBE-RT | 0.193 | 0.863 | 0.223 | 0.823 | (0.00, 1.88) | 0.201 |
| CG-AE-MBE | 0.013 | 0.370 | 0.036 | 0.971 | (0.00, 0.74) | 0.246 |

Note: AE: aerobic exercise; MBE: mind–body exercise; RT: resistance training; MCE: multi-component exercise; FTr: functional training; CG: control group.

# Supplementary 8 Publication bias

Figure 5. Funnel figure

Note: AE: aerobic exercise; MBE: mind–body exercise; RT: resistance training; MCE: multi-component exercise; FT: flexibility training; FTr: functional training; CG: control group.

# Supplementary 9 Sensitivity analyses

Table 6. Eliminate the study results individually

| Excluded study | RT-CG SMD (95% CI) | τ² |
| --- | --- | --- |
| Chen (2023) | 1.52 (0.78, 2.27) | 0.24 |
| Zhu (2018) | 1.52 (0.79, 2.24) | 0.22 |
| Song (2019) | 1.49 (0.79, 2.20) | 0.20 |
| Wei (2014) | 1.51 (0.78, 2.24) | 0.23 |
| Chang (2024) | 1.52 (0.78, 2.25) | 0.24 |
| Ayari (2023) | 1.50 (0.78, 2.23) | 0.22 |
| Yu (2021) | 1.50 (0.79, 2.22) | 0.21 |
| Tao (2019) | 1.48 (0.77, 2.19) | 0.21 |
| Silva-Batista (2016) | 1.98 (1.08, 2.87) | 0.20 |
| Lautenschlager (2008) | 1.50 (0.79, 2.22) | 0.21 |
| Liu (2022) | 1.47 (0.77, 2.17) | 0.19 |
| Lin (2025) | 1.50 (0.76, 2.23) | 0.23 |
| Sánchez-Alcalá (2025) | 1.50 (0.77, 2.23) | 0.23 |
| Yang (2015) | 1.50 (0.78, 2.21) | 0.21 |
| Greblo Jurakic (2017) | 1.45 (0.56, 2.34) | 0.22 |
| Picelli (2016) | 1.51 (0.78, 2.23) | 0.22 |
| Nadeau (2014) | 1.51 (0.79, 2.23) | 0.22 |
| McKee (2013) | 1.53 (0.83, 2.23) | 0.19 |
| Hoffmann (2016) | 1.51 (0.79, 2.23) | 0.21 |
| Holthoff (2015) | 1.15 (0.32, 1.98) | 0.20 |
| Vreugdenhil (2011) | 1.50 (0.78, 2.23) | 0.22 |
| Fajersztajn (2008) | 1.51 (0.78, 2.23) | 0.22 |
| de Oliveira Silva (2019) | 1.50 (0.79, 2.22) | 0.21 |
| Tsai (2025) | 1.49 (0.81, 2.18) | 0.18 |
| Kolk (2019) | 1.50 (0.82, 2.18) | 0.18 |
| Rios Romenets (2015) | 1.52 (0.79, 2.24) | 0.22 |
| Gobbi (2021) | 1.51 (0.77, 2.25) | 0.24 |
| Huang (2024) | 1.50 (0.79, 2.21) | 0.21 |

# Supplementary 10 Subgroup analyses

Table 7. The result of subgroup analyses.

| Moderator | Category | Studies | Hedge’s g (95%CI) | *P* |
| --- | --- | --- | --- | --- |
| Age | ≤60 | 1 | Single study | 0.13 |
|  | 61-70 | 17 | 0.65 (0.41, 0.89) |  |
|  | >70 | 10 | 0.79 (0.29, 1.29) |  |
| Type of disease | MCI | 11 | 0.70 (0.36, 1.04) | 0.24 |
|  | PD | 9 | 0.35 (-0.06, 0.76) |  |
|  | AD | 8 | 0.87 (0.37, 1.38) |  |
| Duration of exercise | ≤6 weeks | 2 | 0.75 (-0.35, 1.84) | 0.60 |
|  | 7-12 weeks | 14 | 0.76 (0.34, 1.17) |  |
|  | >12 weeks | 12 | 0.51 (0.22, 0.79) |  |
| Frequency of exercise (times/week) | 1-2 times | 8 | 0.51 (0.22, 0.80) | 0.74 |
|  | 3-5 times | 19 | 0.70 (0.37, 1.02) |  |
|  | 6-7times | 1 | Single study |  |
| Exercise time (min/time) | ≤30min | 4 | 1.10 (0.33, 1.88) | 0.26 |
|  | 31-60min | 22 | 0.59 (0.34, 0.85) |  |
|  | > 60min | 2 | 0.23 (-0.82, 1.28) |  |

# Supplementary 11 Meta-regression

Table 8. Meta-regression analysis

| Moderator | Coefficient (95 %CI) | SE | *P* | Residual τ² |
| --- | --- | --- | --- | --- |
| Age | 0.31 (-0.12, 0.74) | 0.22 | 0.158 | 0.289 |
| Type of disease | 0.06 (-0.24, 0.35) | 0.15 | 0.713 | 0.317 |
| Duration of exercise | -0.194 (-0.59, 0.20) | 0.20 | 0.340 | 0.300 |
| Frequency of exercise | 0.18 ( -0.30, 0.67) | 0.25 | 0.460 | 0.309 |
| Exercise time | -0.44 (-0.96, 0.08) | 0.26 | 0.096 | 0.284 |

# Supplementary 12 Transitivity assessment

Table 9. The results of transitivity assessment.

| **Modifier** | **Category** | **AE** | **MBE** | **RT** | **MCE** | **FT** | **FTr** |
| --- | --- | --- | --- | --- | --- | --- | --- |
| Age | ≤60 years | 1 | 0 | 0 | 0 | 0 | 0 |
|  | 61–70 years | 10 | 8 | 2 | 2 | 1 | 2 |
|  | >70 years | 4 | 4 | 1 | 2 | 0 | 0 |
| Assessment tools | MoCA | 8 | 10 | 2 | 0 | 0 | 0 |
|  | MMSE | 6 | 2 | 1 | 3 | 0 | 2 |
|  | ADAS-Cog | 1 | 0 | 0 | 1 | 1 | 0 |
| Type of disease | MCI | 6 | 8 | 1 | 0 | 0 | 1 |
|  | PD | 5 | 4 | 1 | 1 | 0 | 1 |
|  | AD | 4 | 0 | 1 | 3 | 1 | 0 |
| Duration of exercise | ≤6 weeks | 2 | 0 | 0 | 0 | 0 | 0 |
|  | 7–12 weeks | 4 | 9 | 3 | 2 | 0 | 0 |
|  | >12 weeks | 9 | 3 | 0 | 2 | 1 | 2 |
| Frequency of exercise | 1–2/week | 1 | 5 | 0 | 3 | 0 | 1 |
|  | 3–5/week | 13 | 7 | 3 | 1 | 1 | 1 |
|  | 6–7/week | 1 | 0 | 0 | 0 | 0 | 0 |
| Exercise time | ≤30 min | 2 | 2 | 1 | 0 | 0 | 1 |
|  | 31–60 min | 13 | 9 | 1 | 4 | 1 | 1 |
|  | >60 min | 0 | 1 | 1 | 0 | 0 | 0 |
| Risk of bias | Low | 8 | 6 | 1 | 1 | 1 | 1 |
|  | Some concerns | 5 | 3 | 1 | 2 | 0 | 1 |
|  | High | 2 | 3 | 1 | 1 | 0 | 0 |

Note: Potential effect modifiers were compared across exercise nodes. Counts represent the number of trial-node records, so multi-arm trials may contribute to more than one exercise node.

Table 10. The results of transitivity assessment.

| Potential effect modifier | Cramér’s V | Association P | Interpretation |
| --- | --- | --- | --- |
| Age | 0.223 | 0.961 | Smaller observed imbalance |
| Assessment tools | 0.574 | 0.007 | Largest imbalance |
| Type of disease | 0.443 | 0.151 | Largest imbalance |
| Duration of exercise | 0.440 | 0.159 | Largest imbalance |
| Frequency of exercise | 0.406 | 0.272 | Smaller observed imbalance |
| Exercise time | 0.383 | 0.371 | Smaller observed imbalance |
| Risk of bias | 0.226 | 0.957 | Smaller observed imbalance |

Table 11. Diagnosis- and risk-of-bias–based sensitivity analyses.

| Sensitivity analysis | Included evidence | RT-CG SMD  (95% CI) | Ranking result |
| --- | --- | --- | --- |
| Primary analysis | All diagnoses | 1.50 (0.77, 2.23) | RT ranked first |
| Exclude AD | PD + MCI | 1.15 (0.29, 2.01) | RT ranked first |
| Exclude PD | AD + MCI | 2.11 (1.26, 2.96) | RT ranked first |
| Exclude MCI | AD + PD | 1.47 (0.50, 2.44) | RT ranked first |
| Restrict to low risk/some concerns | Exclude five high-risk trials | 1.45 (0.57, 2.32) | RT ranked first |

# Supplementary 13 CINeMA certainty-of-evidence assessment

Table 12. The results of CINeMA certainty-of-evidence assessment.

| Comparison | Within-study bias | Reporting bias | Indirectness | Imprecision | Heterogeneity | Incoherence | Overall certainty | Rationale |
| --- | --- | --- | --- | --- | --- | --- | --- | --- |
| AE-CG | Some concerns | Some concerns | Some concerns | Some concerns | Some concerns | No concerns | Low | Downgraded for risk of bias, possible reporting bias, imprecision, and heterogeneity. |
| MBE-CG | Some concerns | Some concerns | No concerns | Some concerns | Some concerns | No concerns | Low | One of the broadest evidence bases among active modalities, but heterogeneity and possible reporting bias remained. |
| RT-CG | Some concerns | Some concerns | No concerns | Some concerns | Major concerns | No concerns | Low | Large estimate and high ranking probability, but only three trials and substantial heterogeneity. |
| MCE-CG | Some concerns | Some concerns | Some concerns | Some concerns | Some concerns | No concerns | Low | Intervention content was heterogeneous and the node included four trials. |
| FT-CG | Some concerns | Major concerns | No concerns | Major concerns | Some concerns | No concerns | Very low | One small trial; wide interval included potentially beneficial and harmful effects. |
| FTr-CG | Some concerns | Major concerns | No concerns | Major concerns | Some concerns | No concerns | Very low | Two heterogeneous trials and wide uncertainty; ranking is unstable. |

**References**

Ayari, S., Abellard, A., Sakrani, S., Krinitskaia, A., Grzelak, M., Nader, R. B., & Gavarry, O. (2023). Comparison of dance and aerobic exercise on cognition and neuropsychiatric symptoms in sedentary older adults with cognitive impairment. *European Geriatric Medicine*, *14*(6), 1289–1299. https://doi.org/10.1007/s41999-023-00849-z

Chang, C.-L., Lin, T.-K., Pan, C.-Y., Wang, T.-C., Tseng, Y.-T., Chien, C.-Y., & Tsai, C.-L. (2024). Distinct effects of long-term Tai Chi Chuan and aerobic exercise interventions on motor and neurocognitive performance in early-stage Parkinson’s disease: A randomized controlled trial. *European Journal of Physical and Rehabilitation Medicine*, *60*(4), 621–633. https://doi.org/10.23736/S1973-9087.24.08166-8

Chen, Y., Qin, J., Tao, L., Liu, Z., Huang, J., Liu, W., Xu, Y., Tang, Q., Liu, Y., Chen, Z., Chen, S., Liang, S., Chen, C., Xie, J., Liu, J., Chen, L., & Tao, J. (2023). Effects of Tai Chi Chuan on Cognitive Function in Adults 60 Years or Older With Type 2 Diabetes and Mild Cognitive Impairment in China: A Randomized Clinical Trial. *JAMA Network Open*, *6*(4), e237004. https://doi.org/10.1001/jamanetworkopen.2023.7004

de Oliveira Silva, F., Ferreira, J. V., Plácido, J., Sant’Anna, P., Araújo, J., Marinho, V., Laks, J., & Camaz Deslandes, A. (2019). Three months of multimodal training contributes to mobility and executive function in elderly individuals with mild cognitive impairment, but not in those with Alzheimer’s disease: A randomized controlled trial. *Maturitas*, *126*, 28–33. https://doi.org/10.1016/j.maturitas.2019.04.217

Fajersztajn, L., Cordeiro, R. C., Andreoni, S., & Garcia, J. T. (2008). Effects of functional physical activity on the maintenance of motor function in Alzheimer’s disease. *Dementia & Neuropsychologia*, *2*(3), 233–240. https://doi.org/10.1590/S1980-57642009DN20300013

Gobbi, L. T. B., Pelicioni, P. H. S., Lahr, J., Lirani-Silva, E., Teixeira-Arroyo, C., & Santos, P. C. R. D. (2021). Effect of different types of exercises on psychological and cognitive features in people with Parkinson’s disease: A randomized controlled trial. *Annals of Physical and Rehabilitation Medicine*, *64*(1), 101407. https://doi.org/10.1016/j.rehab.2020.05.011

Greblo Jurakic, Z., Krizanic, V., Sarabon, N., & Markovic, G. (2017). Effects of feedback-based balance and core resistance training vs. Pilates training on cognitive functions in older women with mild cognitive impairment: A pilot randomized controlled trial. *Aging Clinical and Experimental Research*, *29*(6), 1295–1298. https://doi.org/10.1007/s40520-017-0740-9

Hoffmann, K., Sobol, N. A., Frederiksen, K. S., Beyer, N., Vogel, A., Vestergaard, K., Brændgaard, H., Gottrup, H., Lolk, A., Wermuth, L., Jacobsen, S., Laugesen, L. P., Gergelyffy, R. G., Høgh, P., Bjerregaard, E., Andersen, B. B., Siersma, V., Johannsen, P., Cotman, C. W., … Hasselbalch, S. G. (2016). Moderate-to-High Intensity Physical Exercise in Patients with Alzheimer’s Disease: A Randomized Controlled Trial. *Journal of Alzheimer’s Disease: JAD*, *50*(2), 443–453. https://doi.org/10.3233/JAD-150817

Holthoff, V. A., Marschner, K., Scharf, M., Steding, J., Meyer, S., Koch, R., & Donix, M. (2015). Effects of Physical Activity Training in Patients with Alzheimer’s Dementia: Results of a Pilot RCT Study. *PLoS ONE*, *10*(4), e0121478. https://doi.org/10.1371/journal.pone.0121478

Huang, Y., Ou, H., Zhao, W., Lin, Q., Xue, Y., Xia, R., Tan, Z., Zhao, X., Xiong, L., Yan, Z., Zheng, Z., & Wen, J. (2024). The effects of moderate-intensity aerobic exercise on cognitive function in individuals with stroke-induced mild cognitive impairment: A randomized controlled pilot study. *Journal of Rehabilitation Medicine*, *56*, jrm33001. https://doi.org/10.2340/jrm.v56.33001

Kolk, N. M. van der, Vries, N. M. de, Kessels, R. P. C., Joosten, H., Zwinderman, A. H., Post, B., & Bloem, B. R. (2019). Effectiveness of home-based and remotely supervised aerobic exercise in Parkinson’s disease: A double-blind, randomised controlled trial. *The Lancet Neurology*, *18*(11), 998–1008. https://doi.org/10.1016/S1474-4422(19)30285-6

Lautenschlager, N. T., Cox, K. L., Flicker, L., Foster, J. K., van Bockxmeer, F. M., Xiao, J., Greenop, K. R., & Almeida, O. P. (2008). Effect of physical activity on cognitive function in older adults at risk for Alzheimer disease: A randomized trial. *JAMA*, *300*(9), 1027–1037. https://doi.org/10.1001/jama.300.9.1027

Lin, L., He, Y.-X., Wen, Q., Liu, J.-Y., Dai, Y., Fei, Y.-Z., Li, H., Li, C.-Q., & Zhou, H. (2025). Evaluation of the efficacy of Tai Chi on the cognitive function of patients with mild cognitive dysfunction and research on its mechanism. *Frontiers in Aging Neuroscience*, *17*, 1435996. https://doi.org/10.3389/fnagi.2025.1435996

Liu, H.-H., Wang, R.-Y., Cheng, S.-J., Liao, K.-K., Zhou, J.-H., & Yang, Y.-R. (2022). Effects of square-stepping exercise on executive function in individuals with Parkinson’s disease: A randomized controlled pilot study. *Geriatric Nursing*, *47*, 273–279. https://doi.org/10.1016/j.gerinurse.2022.08.004

McKee, K. E., & Hackney, M. E. (2013). The Effects of Adapted Tango on Spatial Cognition and Disease Severity in Parkinson’s Disease. *Journal of Motor Behavior*, *45*(6), 10.1080/00222895.2013.834288. https://doi.org/10.1080/00222895.2013.834288

Nadeau, A., Pourcher, E., & Corbeil, P. (2014). Effects of 24 wk of Treadmill Training on Gait Performance in Parkinson’s Disease. *Medicine & Science in Sports & Exercise*, *46*(4), 645. https://doi.org/10.1249/MSS.0000000000000144

Picelli, A., Varalta, V., Melotti, C., Zatezalo, V., Fonte, C., Amato, S., Saltuari, L., Santamato, A., Fiore, P., & Smania, N. (2016). Effects of treadmill training on cognitive and motor features of patients with mild to moderate Parkinson’s disease: A pilot, single-blind, randomized controlled trial. *Functional Neurology*, *31*(1), 25–31. https://doi.org/10.11138/fneur/2016.31.1.025

Rios Romenets, S., Anang, J., Fereshtehnejad, S.-M., Pelletier, A., & Postuma, R. (2015). Tango for treatment of motor and non-motor manifestations in Parkinson’s disease: A randomized control study. *Complementary Therapies in Medicine*, *23*(2), 175–184. https://doi.org/10.1016/j.ctim.2015.01.015

Sánchez-Alcalá, M., Aibar-Almazán, A., Carcelén-Fraile, M. D. C., Castellote-Caballero, Y., Cano-Sánchez, J., Achalandabaso-Ochoa, A., Muñoz-Perete, J. M., & Hita-Contreras, F. (2025). Effects of Dance-Based Aerobic Training on Frailty and Cognitive Function in Older Adults with Mild Cognitive Impairment: A Randomized Controlled Trial. *Diagnostics*, *15*(3), 351. https://doi.org/10.3390/diagnostics15030351

Silva-Batista, C., Corcos, D. M., Roschel, H., Kanegusuku, H., Gobbi, L. T. B., Piemonte, M. E. P., Mattos, E. C. T., DE Mello, M. T., Forjaz, C. L. M., Tricoli, V., & Ugrinowitsch, C. (2016). Resistance Training with Instability for Patients with Parkinson’s Disease. *Medicine and Science in Sports and Exercise*, *48*(9), 1678–1687. https://doi.org/10.1249/MSS.0000000000000945

Song, D., & Yu, D. S. F. (2019). Effects of a moderate-intensity aerobic exercise programme on the cognitive function and quality of life of community-dwelling elderly people with mild cognitive impairment: A randomised controlled trial. *International Journal of Nursing Studies*, *93*, 97–105. https://doi.org/10.1016/j.ijnurstu.2019.02.019

Tao, J., Liu, J., Chen, X., Xia, R., Li, M., Huang, M., Li, S., Park, J., Wilson, G., Lang, C., Xie, G., Zhang, B., Zheng, G., Chen, L., & Kong, J. (2019). Mind-body exercise improves cognitive function and modulates the function and structure of the hippocampus and anterior cingulate cortex in patients with mild cognitive impairment. *NeuroImage. Clinical*, *23*, 101834. https://doi.org/10.1016/j.nicl.2019.101834

Tsai, C.-L., Chien, C.-Y., Pan, C.-Y., Tseng, Y.-T., Wang, T.-C., & Lin, T.-K. (2025). Effects of long-term Tai Chi vs. Aerobic exercise on antioxidant activity and cognitive function in individuals with Parkinson’s disease. *Behavioural Brain Research*, *476*, 115274. https://doi.org/10.1016/j.bbr.2024.115274

Vreugdenhil, A., Cannell, J., Davies, A., & Razay, G. (2012). A community-based exercise programme to improve functional ability in people with Alzheimer’s disease: A randomized controlled trial. *Scandinavian Journal of Caring Sciences*, *26*(1), 12–19. https://doi.org/10.1111/j.1471-6712.2011.00895.x

Wei, X., & Ji, L. (2014). Effect of handball training on cognitive ability in elderly with mild cognitive impairment. *Neuroscience Letters*, *566*, 98–101. https://doi.org/10.1016/j.neulet.2014.02.035

Yang, S.-Y., Shan, C.-L., Qing, H., Wang, W., Zhu, Y., Yin, M.-M., Machado, S., Yuan, T.-F., & Wu, T. (2015). The Effects of Aerobic Exercise on Cognitive Function of Alzheimer’s Disease Patients. *CNS & Neurological Disorders Drug Targets*, *14*(10), 1292–1297. https://doi.org/10.2174/1871527315666151111123319

Yu, F., Vock, D. M., Zhang, L., Salisbury, D., Nelson, N. W., Chow, L. S., Smith, G., Barclay, T. R., Dysken, M., & Wyman, J. F. (2021). Cognitive Effects of Aerobic Exercise in Alzheimer’s Disease: A Pilot Randomized Controlled Trial. *Journal of Alzheimer’s Disease: JAD*, *80*(1), 233–244. https://doi.org/10.3233/JAD-201100

Zhu, Y., Wu, H., Qi, M., Wang, S., Zhang, Q., Zhou, L., Wang, S., Wang, W., Wu, T., Xiao, M., Yang, S., Chen, H., Zhang, L., Zhang, K. C., Ma, J., & Wang, T. (2018). Effects of a specially designed aerobic dance routine on mild cognitive impairment. *Clinical Interventions in Aging*, *13*, 1691–1700. https://doi.org/10.2147/CIA.S163067
